# Mutational Landscape of Patients Referred for Elevated Hemoglobin Level

**DOI:** 10.3390/curroncol29100568

**Published:** 2022-09-30

**Authors:** Pratibha Bhai, Benjamin Chin-Yee, Victor Pope, Ian Cheong, Maxim Matyashin, Michael A. Levy, Aidin Foroutan, Alan Stuart, Cyrus C. Hsia, Hanxin Lin, Bekim Sadikovic, Ian Chin-Yee

**Affiliations:** 1 Molecular Diagnostic Division, London Health Sciences Centre, London, ON N6A 5W9, Canada; 2 Verspeeten Clinical Genome Centre, London Health Sciences Centre, London, ON N6A 5W9, Canada; 3 Division of Hematology, Department of Medicine, Schulich School of Medicine and Dentistry, Western University, London, ON N6A 5W9, Canada; 4 Division of Hematology, Department of Medicine, London Health Sciences Centre, London, ON N6A 5W9, Canada; 5 Department of Pathology & Laboratory Medicine, Schulich School of Medicine and Dentistry, Western University, London, ON N6A 5W9, Canada

**Keywords:** erythrocytosis, polycythemia vera, JAK2, myeloid

## Abstract

*Background:* Since the identification of *JAK2 V617F* and exon 12 mutations as driver mutations in polycythemia vera (PV) in 2005, molecular testing of these mutations for patients with erythrocytosis has become a routine clinical practice. However, the incidence of myeloid mutations other than the common *JAK2 V617F* mutation in unselected patients referred for elevated hemoglobin is not well studied. This study aimed to characterize the mutational landscape in a real-world population of patients referred for erythrocytosis using a targeted next-generation sequencing (NGS)-based assay. *Method:* A total of 529 patients (hemoglobin levels >160 g/L in females or >165 g/L in males) were assessed between January 2018 and May 2021 for genetic variants using the Oncomine Myeloid Research Assay (ThermoFisher Scientific, Waltham, MA, USA) targeting 40 key genes with diagnostic and prognostic implications in hematological conditions (17 full genes and 23 genes with clinically relevant “hotspot” regions) and a panel of 29 fusion driver genes (>600 fusion partners). *Results:* *JAK2* mutations were detected in 10.9% (58/529) of patients, with 57 patients positive for *JAK2 V617F*, while one patient had a *JAK2* exon 12 mutation. Additional mutations were detected in 34.5% (20/58) of *JAK2*-positive patients: *TET2* (11; 19%), *DNMT3A* (2;3.4%), *ASXL1* (2; 3.4%), *SRSF2* (2; 3.4%), *BCOR* (1; 1.7%), *TP53* (1; 1.7%), and *ZRSR2* (1; 1.7%). Diagnosis of PV was suspected in 2 *JAK2*-negative patients based on the 2016 World Health Organization (WHO) diagnostic criteria. Notably, one patient carried mutations in the *SRSF2* and *TET2* genes, and the other patient carried mutations in the *SRSF2, IDH2,* and *ASXL1* genes. Three *JAK2*-negative patients with elevated hemoglobin who tested positive for *BCR/ABL1* fusion were diagnosed with chronic myeloid leukemia (CML) and excluded from further analysis. The remaining 466 *JAK2*-negative patients were diagnosed with secondary erythrocytosis and mutations were found in 6% (28/466) of these cases. *Conclusion:* Mutations other than *JAK2* mutations were frequently identified in patients referred for erythrocytosis, with mutations in the *TET2, DNMT3A,* and *ASXL1* genes being detected in 34.5% of *JAK2*-positive PV patients. The presence of additional mutations, such as *ASXL1 mutations,* in this population has implications for prognosis. Both the incidence and mutation type identified in patients with secondary erythrocytosis likely reflects incidental, age-associated clonal hematopoiesis of indeterminate potential (CHIP).

## 1. Introduction

Elevated hemoglobin is a common reason for referral to hematologists, primarily to exclude polycythemia vera (PV), which, if left untreated, often has high morbidity and mortality. *JAK2 V617F* (in exon 14) and exon 12 mutations are major diagnostic criteria for PV, found in 99% of confirmed cases [1]. Traditional methods for testing *JAK2* mutations, including qualitative and quantitative PCR-based methods and Sanger sequencing, have been in use in clinical practice since the identification of these driver mutations in 2005 [2,3]. The allele burden of JAK2 V617F has been shown to be associated with the phenotypic presentation of the disease; therefore, sensitive molecular techniques, such as quantitative polymerase chain reaction (qPCR) and droplet digital PCR (ddPCR), are used for a quantitative evaluation of JAK2 V617F mutation allele burden for diagnostic purposes and MRD monitoring [4]. Although these targeted methods can provide rapid and reliable results, they are limited in their capacities to concomitantly identify additional mutations that might influence diagnosis and prognosis in both *JAK2*-positive PV and the rare *JAK2*-negative patients [5]. In recent years, the introduction of high-throughput Next Generation Sequencing (NGS) has transformed molecular testing for hematological malignancies [6]. Simultaneous screening of multiple genes provides an opportunity to discover novel genetic mutations in genes which hold diagnostic, prognostic, and therapeutic relevance.

These NGS panels target genes that are associated with multiple hematological conditions, e.g., *DNMT3A*, *TET2*, *ASXL1,* and genes that are implicated in the pathogenesis of specific hematological disorders, e.g., *JAK2* for myeloproliferative neoplasms (MPN). Targeted panel-based testing is now routinely performed for many hematological diseases including acute myeloid leukemia (AML), myelodysplastic syndromes (MDS), MPN, and lymphoproliferative disorders [7,8]. However, the incidence of additional somatic mutations other than *JAK2* mutations in unselected patients referred for elevated hemoglobin is less well studied. Our laboratory has previously described the clinical validation and implementation of an NGS pipeline combining a DNA sequencing and RNA-based gene fusion panel for the clinical diagnosis of several hematological diseases [7,9]. In the present study, we aimed to characterize the spectrum of mutations in patients referred for elevated hemoglobin using this NGS-based assay, targeting genes with diagnostic and prognostic relevance in hematological conditions.

## 2. Materials and Methods

### 2.1. Subjects (Patient Selection Criteria)

This study was conducted at London Health Sciences Centre, which serves a population of 2.5 million in southwestern Ontario, Canada. Patients referred for elevated hemoglobin levels between January 2018 and May 2021 were included in this study. Elevated hemoglobin levels were defined as (>160 g/L in females or >165 g/L in males). Patient demographics, laboratory data, and final diagnosis were extracted from the electronic medical record. For all patients with genetic mutations, clinical diagnosis was confirmed by three independent hematologists.

### 2.2. Next Generation Sequencing Assay

All patients underwent molecular testing using the NGS-based Oncomine Myeloid Research Assay (ThermoFisher Scientific, Waltham, MA, USA), which has been previously validated in our laboratory and implemented at our centre [7,9]. This assay involves analyzing DNA for sequence variants in 40 key target genes (17 genes with full coverage and 23 genes with partial coverage, including clinically relevant “hotspot” regions) and RNA for 29 gene fusions including 687 fusion partners associated with hematologic disorders. This assay sequences exon 12–15 of the JAK2 gene. DNA/RNA extraction, library preparation, template preparation, sequencing, variant (DNA and RNA fusion) calling, and data analyses are performed as previously described [9]. Variants are classified and interpreted by Genome Analysts, certified clinical molecular geneticists, and/or molecular pathologists following the AMP/ASCO/CAP joint guideline [10]. All variants identified at a variant allele frequency (VAF) ≥5% were assessed and classified. Variants were classified into four tiers (Tier I to IV) based on their level of clinical significance in cancer diagnosis, prognosis, and/or therapeutics. Variants classified as Tier I and II were of strong clinical significance, Tier III variants were with unknown clinical significance due to lack of significant evidence, and Tier IV variants were benign or likely benign. Only Tier I and II variants at VAF ≥5% were reported and further analyzed in this study.

To further characterize the mutational landscape of erythrocytosis, a literature search was performed to summarize recently reported mutations in patients with non-MPN erythrocytosis. A hand search through abstracts and non-peer-reviewed reports (“grey literature”) was conducted. Articles were included if they reported genetic analysis of patients with erythrocytosis where MPN and secondary causes were ruled out. A total of 741 articles (within a period from July 2017 to July 2022) were found, and 19 [11,12,13,14,15,16,17,18,19,20,21,22,23,24,25,26,27,28,29,30] of these were included in our summary. The results are summarized in Supplementary File II (Summary of mutations associated with MPN negative erythrocytosis reported in literature between July 2017 and July 2022).

## 3. Results

### 3.1. Patient Cohort

A total of 529 patients with erythrocytosis (389 males and 140 females) were included in this study. The demographics and clinical characteristics of these patients are summarized in Table 1. Out of 529 patients, 60 patients were diagnosed with polycythemia vera, 3 with chronic myeloid leukemia, and the remaining 466 patients were diagnosed with secondary erythrocytosis. A secondary diagnosis was confirmed for patients when the cause of the abnormal blood count was not due to intrinsic blood or marrow problem but due to (secondary) other causes, including medications and underlying chronic diseases, etc. as confirmed by the independent chart review done by a hematologist for each patient. In clinical practice, the diagnosis of secondary erythrocytosis is most often made based on clinical assessment and judgment based on the exclusion of PV (mutation analysis, EPO levels, and marrow) along with risk factors for secondary causes. We chose this pragmatic clinical approach in assigning patients a diagnosis of a secondary cause for elevated hemoglobin as it best reflects actual clinical practice where every possible rare cause of secondary erythrocytosis cannot be excluded.

### 3.2. Tier I/II Variants Identified in JAK2-Positive Patients with Erythrocytosis

**Figure 1:**
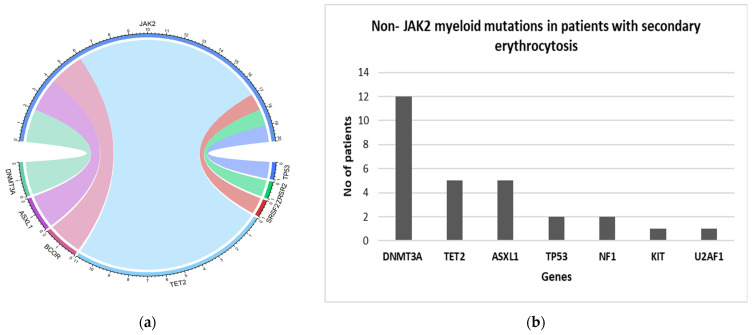
(**a**) Circos plots showing co-occurrence of additional mutation in JAK2 positive PV patients; (**b**) Bar Graph showing myeloid mutations in JAK2 negative patients with secondary erythrocytosis.

A total of 92 patients tested positive for Tier I/II variants by NGS. Mutations in the *JAK2* gene were detected in 10.9% (58/529) of patients, all of whom had a confirmed diagnosis of PV. The majority of *JAK2*-mutated PV patients (*n* = 57) were positive for *JAK2 V617F*, while one patient had an exon 12 mutation. In patients with JAK2-positive PV the variant allele frequency was >10% for all patients (Supplementary File I (Variants identified by NGS in this study)). Mutations in other genes were detected in 34.5% (20/58) of *JAK2*-positive patients (Table 2 and Figure 1a). Among these, mutations in the *TET2* gene are the most common, followed by mutations in the *DNMT3A* and *ASXL1* gene.

### 3.3. Tier I/II Variants Identified in JAK2-Negative Patients

Interestingly, two *JAK2*-negative patients were clinically diagnosed as suspected PV based on the 2016 WHO diagnostic criteria by their treating physicians. Both patients were found to have non-*JAK2* mutations. One patient, 73 years of age, had a variant in *SRSF2* and *TET2* genes. The other patient, 72 years of age, had one variant in *SRSF2, IDH2, and ASXL1* genes. Three *JAK2*-negative patients were found to carry a *BCR/ABL1* fusion and were diagnosed as CML. These *JAK2*-negative suspected PV patients and CML patients were excluded from further analysis. Other than *BCR::ABL1*, no gene fusions were detected in any other patient.

A total of 466 *JAK2*-negative patients were diagnosed to have secondary erythrocytosis. Tier I/II variants of multiple genes were found in 6% (28/466) of these cases (Table 2 and Figure 1b). Among these, DNMT3A is the most commonly mutated gene (in 2.6% cases). This is followed by *TET2* and *ASXL1* gene, which occurred in ~1% cases. All patients with *JAK2*-negative secondary erythrocytosis had only one myeloid gene mutation detected.

### 3.4. Tier III Variants (Unknown Significance)

A total of 119 patients were found to carry 136 Tier III variant (s) or variants of unknown significance. Of these, 9 patients also carried *JAK2* V617F and 10 patients also had Tier I/II variants in other genes. One hundred patients had tier III variants only. Out of the 136 Tier III variants, 8 were observed in more than one patient. Tier III variants were frequently observed in *SH2B3* (4%), *TET2* (3%), *BCOR* (2.5%), *ASXL1* (2.7%), *DNMT3A* (1.9%), and *NF1* (2.1%) genes in patients with secondary erythrocytosis. The majority (>90%) of these variants were observed at a VAF (variant allele frequency) near to 50% or 100%, suggesting that they are either heterozygous or homozygous variants 4.

## 4. Discussion

Erythrocytosis is a common reason for referral to hematologists. Distinguishing between PV and secondary causes of erythrocytosis often relies on molecular diagnostics [31]. Although *JAK2 V617F* and exon 12 mutations are well described in PV, the genomic landscape is evolving with the description of additional mutations that impact prognosis and risk stratification [32] and may help guide future management. The incidence of mutations in genes associated with hematological conditions in unselected patients referred for elevated hemoglobin, however, is not as well documented. Our centre integrated NGS-based testing of a wide spectrum of genes associated with hematological malignancies into the existing diagnostic algorithms for the analysis of hematologic malignancies.

In this study, we investigated the mutational profiles in 529 patients with erythrocytosis and found clinically relevant mutations (Tier I/II) in 17.3% (92/529) of them. As expected, *JAK2* was the most common mutation observed in 10.9% of patients who were diagnosed with PV. Additional mutations were identified in 34.5% of these *JAK2*-positive PV patients, with the highest frequencies observed in the *TET2* gene (19%), followed by *DNMT3A, ASXL1,* and *BCOR* genes (3.4%). In previous studies, mutations additional to *JAKV 617F* have been frequently reported in PV patients (53–78%) in a variety of genes, with maximum frequency observed in the *TET2* (22–30%) gene, followed by *ASXL1*(10 to 12%) and *DNMT3A* gene (13%) [32,33,34]. In a recent study by Wouters et al., 38% (51/133) of patients with erythrocytosis (defined by strict criteria described by McMullin et al.) [35] were positive for a somatic mutation, and in our study, 17.3% (92/529) patients with erythrocytosis defined by (wide WHO criteria) tested positive by NGS [15]. In this study, the number of patients with more than 1 mutation is higher (55%; 28/51 vs. 24%; 22/92 in our study), but interestingly the JAK2 mutation rate was higher in our study (10.9% vs. 5.3%). CHIP-related mutations rate was lower (6% vs. 18%) but the most commonly mutated CHIP genes were similar in the two studies (DNMT3A, TET2, and ASXL1). However, the frequency of BCOR variants (0.3%) was not significantly high in our study as reported by Wouters et al. (15%) [15]. The difference in the mutation rates in comparison with other reported studies could be attributed to differences in referral patterns and patient selection criteria, study population, coverage, and gene content of the NGS assay, and variant assessment and interpretation criteria. Analysis of somatic mutations in our populations based on other definitions of erythrocytosis, such as those by McMullin et al. [35], would be informative but were not performed. Specifically, in our study, only Tier I/II variants, classified based on the guidelines recommended by ASCO/AMP/CAP [9] were reported whereas other reports have included variants now classified as Tier III. The presence of variants in additional genes has been associated with predicting overall survival and leukemic transformation in patients with myeloproliferative neoplasm including PV [36]. *ASXL1* gene mutations, as an independent prognostic marker and also in combination with age and vascular complications, are associated with inferior survival in PV patients. *SRSF2* gene mutations are associated with an increased risk of leukemic transformation and reduced overall survival in *JAK2*-mutated PV patients. Interestingly, mutations in the *SRSF2* gene were detected in three patients in our cohort; one was *JAK2 V617F*-positive and two were *JAK2*-negative. One of the *JAK2*-negative suspected PV patients also had *IDH2* and *ASXL1* gene mutation in addition to *SRSF2*, which are further linked with inferior overall survival and leukemic transformation in *JAK2*-mutated PV [33,37]. Whether the combination of *SFRS2, ASLX1*, and *IDH2* mutations, in the absence of a *JAK2* mutation, is sufficient to confer the MPN phenotype, or describes a unique MPN, is uncertain and warrants further study. Both patients, aged 72 and 73 years, with *JAK2*-negative suspected PV, had typical features of myeloproliferative disorders, including splenomegaly, and required repeated phlebotomy and hydroxyurea therapy. The prognostic utility of these additional mutations identified in patients with suspected *JAK2*-negative PV supports the role of NGS-based genetic testing for patients, and may also be helpful in elucidating other myeloid drivers for the rare patients with *JAK2*-negative PV, a poorly characterized subgroup requiring further research.

The nature and overall incidence of mutations in *JAK2*-negative patients with secondary erythrocytosis (6%) is comparable to the reported incidence of Clonal Hematopoiesis of Indeterminate Potential (CHIP) (4.3%; 746/17152 in all age groups), which indicates that these may represent incidental age-related mutations [38]. While co-occurring mutations in patients with PV have been described in literature, further studies are needed to investigate the significance of variants identified in *JAK2*-negative patients classified as secondary erythrocytosis, to determine if these mutations contribute to clinical phenotype or constitute background CHIP. A review of the literature on recently reported mutations in patients with non-MPN erythrocytosis, highlighted the role of some known genes associated with familial erythrocytosis, such as *EPO, HBB, VHL EGLN1, EPAS1,* and *EPOR*. Some of the less well-studied genes that have come up in the literature review are *SH2B3, HFE, BPGM*, and other CHIP-associated genes, including *DNMT3A, TET2, BCOR*, *and ASXL1.*

We also investigated Tier III variants, or variants of undetermined significance (VUSs), identified in JAK2-negative patients with erythrocytosis. These variants had insufficient evidence as being clinically relevant either due to lack of functional evidence and/or being observed in the general population at <1%. Notably, 4% (19/466) of JAK2-negative patients had variants of unknown significance in the *SH2B3* gene, which may hold relevance in patients with erythrocytosis as a negative regulator of normal hematopoiesis [39]. We are further investigating the potential clinical significance of specific Tier III *SH2B3* variants in another ongoing study.

In this study we explored the mutation spectrum of patients with erythrocytosis using targeted myeloid NGS assay, and found that genetic variants in genes other than *JAK2* are frequently observed in patients with erythrocytosis. However, the precise role of these variants is not clear and further studies are warranted to understand the clinical impact in these patients.

## Figures and Tables

**Table 1:** Patients with erythrocytosis.

	Total Patients	Male	Females
Total Patients, number (%)	529 (100)	390 (73.7)	139 (26.3)
Age; median (range) years	60.6 (18–95)	60.4 (18–95)	61.6 (24–85)
* Height; median (range) cm	173 (125–198)	177 (125–198)	160.5 (141–180)
° Weight; median (range) kg	91.4 (42–227.7)	96.1(52.7–227.7)	79.1 (42–150.3)
** BMI; median (range) kg/m^2^	30.5 (14.9–68.7)	30.9 (17.9–68.7)	30.4 (14.9–53.8)
Hemoglobin; median (range) g/L	175 (160–240)	178 (165–240)	167 (160–217)
Leukocytes; median (range) × 10^9^/L	8.3 (2.2–43.6)	8 (2.2–43.6)	8.8 (4.4–31.7)
Platelet count; median (range) × 10^9^/L	232.5 (31–1498)	220 (31–1161)	278 (50–1498)
Hematocrit; median (range) L/L	0.52 (0.45–0.73)	0.53 (0.46–0.73)	0.51 (0.45–0.67)
^#^ EPO levels; median (range) mIU/mL	8 (1.1–456.1)	8.1 (1.1–456.1)	8 (1.2–111.7)

* Height was recorded for 436/529 patients; ° Weight recorded for 453/529 patients; ** BMI recorded for 436/529 patients; ^#^ Erythropoietin (EPO) levels recorded for 364 patients. mIU/mL—milli-international units per milliliter.

**Table 2:** Mutations identified in *JAK2* positive and negative cohorts.

.	**Total Patients** ***n* = 529 (%)**	**JAK2 Positive Cohort** ***n* = 58 (%)**	**JAK2 Negative Cohort** ***n* = 471(%)**
Hemoglobin; median (range) g/L	175 (160–240)	174.5(161–217)	175(160–240)
Leukocytes; median (range) × 10^9^/L	8.3(2.2–43.6)	10.9(4.3–31.7)	8(2.2–43.6)
Platelet count; median (range) × 10^9^/L	232.5(31–1498)	602.5(50–1498)	224(31–566)
Hematocrit; median (range) L/L	0.52(0.45–0.73)	0.54(0.48–0.67)	0.52(0.45–0.73)
EPO levels; median (range) mIU/mL	8(1.1–456.1)	1.7(1.1–6.5)	8.6(1.3–456.1)
Positive for Tier I/II variants by NGS	91 (17.2)	58	33 (7)
*JAK2 V617F*	57 (10.7)	57 (98.3)	0
*JAK2 exon 12* mutation	1 (0.2)	1 (1.7)	0
Clinical Diagnosis			
Diagnosis of PV *	60	58 (100)	2 ^$^ (0.4)
Diagnosis of CML	3	0	3 (0.6)
Secondary erythrocytosis	466	0	466 (99.0)
Non-*JAK2* mutations	Total Patients *n* = 529 (%)	JAK2 positive cohort *n* = 58 (%)	JAK2 negative with secondary erythrocytosis *n* = 466 (%)
*TET2*	16 (3)	11 (19.0)	5 (1.1)
*DNMT3A*	14 (2.6)	2 (3.4)	12 (2.5)
*ASXL1*	7 (1.3)	2 (3.4)	5 (1.1)
*BCOR*	2 (0.3)	2 (1.7)	0
*SRSF2*	1 (0.2)	1 (1.7)	0
*TP53*	1(0.2)	1 (1.7)	2 (0.4)
*ZRSR2*	1(0.2)	1	0
*NF1*	2 (0.3)	0	2 (0.4)
*KIT*	1 (0.2)	0	1 (0.2)
*U2AF1*	1(0.2)	0	1 (0.2)
Total non-*JAK2* mutations	48 (9)	20 (34.5)	28 (6)

* Diagnosis of polycythemia vera defined by the World Health Organization 2017 classification; ^$^ Out of 2 *JAK2* negative patients with suspected PV, one patient had variants in *SRSF2, TET2* gene and the other had variants in *SRSF2, IDH2, ASXL1* gene CML—chronic myeloid leukemia; NGS—next generation sequencing.

## Data Availability

All data generated or analyzed during this study are included in this published article and its supplementary information files.

## Supplementary Materials

The following supporting information can be downloaded at: https://www.mdpi.com/article/10.3390/curroncol29100568/s1, Supplementary File I: Variants identified by NGS in this study; Supplementary File II: Summary of mutations associated with MPN negative erythrocytosis reported between July 2017 and July 2022.
