# Supplementary material for: Mutational Landscape of Patients Referred for Elevated Hemoglobin Level
Source: Curr Oncol. 2022 Sep 30;29(10):7209–17. doi: 10.3390/curroncol29100568 (PMC9600330; doi:10.3390/curroncol29100568)
Supplement: Supplementary file 1 [file curroncol-29-00568-s001.zip › Supplementary File II-layout.pdf]

Current Oncology

Supplementary Materials

Mutational Landscape of Patients Referred for Elevated Hemoglobin Level

Pratibha Bhai [1,2], Benjamin Chin-Yee [3,4], Victor Pope [3,4], Ian Cheong [5], Maxim Matyashin [3,4], Michael A Levy [1,2,], Aidin Foroutan [1,2,5], Alan Stuart [1,2], Cyrus C Hsia [3,4], Hanxin Lin [1,2,5], Bekim Sadikovic [1,2,5,*] and Ian Chin-Yee [3,4,*]

Table S1. Summary of mutations associated with MPN negative erythrocytosis reported between July 2017 and July 2022.

Article	**N**<br>N mut.[1]	**Method**<br>Gene	Mutation	Inher.[2]
Zmajkovic et al. 2018 [11]	18	NGS of 215 genes		
	10	EPO	c.32delG	AD[3]
Tavakoli et al. 2021 [12]	1	Not specified		
	1	HBB	c.104T>C, p.(Val34Ala)	
Negro et al. 2020 [13]	1	NGS		
	1	VHL	c.598C>T, p.(Arg200Trp)	Het[4]
		TMEM 127	c.268G>A, p.(Val90Met)	Het[4]
Doma et al. 2021 [14]	1	NGS of 24 genes		
	1	EPAS1	c.1609G>A, p.(Gly537Arg)	
Wouters et al. 2020 [15]	133	NGS of 25 genes		
	19	DNMT3 A		
	8	TET2		
	6	ASXL1		
	13	BCOR		
	8	BCORL1		
Núñez-Martínez et al. 2021 [16]	1	Molecular testing of proband’s mother, complete sequencing of VHL		
	1	VHL	exon3del + c.416C>G, p.(Ser139Cys)	cHet[5]
Kristan et al. 2021a [17]	3	NGS of 39 genes		
	2	EGLN1	c.471G>C, p.(Gln157His)	AD[3]
		VHL	c.2572-13A>G	AD[3]
Lenglet et al. 2018 [18]	9	Whole genome sequencing		
	3	VHL	c.598C>T/c.340 + 770T>C, VHL p.(Arg200Trp)/ X1 p.(Ser179Pro?)	Het[4]
	2		c.429C>T, VHL p.(Asp143Asp)	Homo[6]

	1		c.598C>T/c.340 + 694_711dup, VHL p.(Arg200Trp)/ X1 p.(Trp159X?)	Het[4]
	1		c.430G>A/c.340 + 694_711dup, VHL p.(Gly144Arg)/ X1 p.(Trp159X?)	Het[4]
	1		c.340+816A>C, X1 p.(*194Serext*24)	Homo[6]
	1		c.429C>T/c.340 + 770T>C, VHL p.(Asp143Asp)/ X1 p.(Ser179Pro?)	Het[4]
Kristan et al. 2021 [19]	25	NGS of 39 genes		
	1	EPAS1	c.1609G>A, p.(Gly537Arg)	Het[4]
	1		c.2120A>C, p.(Lys707Thr)	Het[4]
	1	JAK2	c.1767C>A, p.(Asn589Lys)	Het[4]
	1	SH2B3	c.901G>A, p.(Glu301Lys)	Het[4]
Gangat et al. 2021 [20]	1	EPOR		
	1	EPOR	c.1316G>A, p.(Trp439*)	AD[3]
Sinnema et al. 2018 [21]	4	Whole exome sequencing		
	2	EGLN1	c.124T.C, p.(Cys42Arg)	AR[7]
Pasquier et al. 2018 [22]	1	EPOR		
	1	EPOR	c.1300dup, p.(Gln434Profs*11)	
Jalowiec et al. 2021 [23]	8	NGS of 13 genes		
	1	EPO	c.*656G>A	AD[3]
	1	EGLN1	c.1088T>G p.(Leu363Arg)	AD[3]
	1		c.122_124delACT, p.(Tyr41del)	AD[3]
	1	VHL	c.340+648T>C	
	1	EPAS1	c.466G>T, p.(Gly156Trp)	AD[3]
	1	JAK2	c.1711G>A, p.(Gly571Ser)	AD[3]
	1		c.1169C>T, p.(Pro390Leu)	AD[3]
	1	SH2B3	c.107C>A, p.(Ala36Glu)	AD[3]
Nabhani et al. 2020 [24]	1	Not specified		
	1	HBB	c.258T>G, p.(Phe85Leu)	
Loganathan et al. 2022 [25]	42	EPAS1, EPOR, VHL, PHD2 exons		
	1	EPAS1	c.1715A>G, p.(Gln572Arg)	Het[4]
	1		c.1694G>T, p.(Ser565Ile)	Het[4]
	1		c.1634T>C, p.(Ile545Thr)	Het[4]
	1		c.1771C>G, p.(Gln591Glu)	Het[4]
	1		c.1859G>A, p.(Cys620Tyr)	Het[4]
	1		c.1969C>T, p.(Gln657*)	Het[4]
	1	EPOR	c.1183G>C, p.(Val395Leu)	Het[4]
	1		c.1028A>C, p.(Gln343Pro)	Het[4]
Chandrasekhar et al. 2020 [26]	65	EPAS1, EPOR, VHL, EGLN1 exons		

	6	EPOR	c.980del, p.(Pro327Hisfs*68)	Het[4]
	3		c.1006G>T, p.(Glu336*)	Het[4]
	2		c.1253G>C, p.(Gly418Ala)	Het[4]
	2		c.1436del, p.(Gly479Alafs*37)	Het[4]
	2		c.1169G>C, p.(Gly390Ala)	Het[4]
	1		c.1231G>A, p.(Ala411Thr)	Het[4]
	1		c.1424G>T, p.(Gly475Val)	Het[4]
	1		c.1470G>C, p.(Glu490Asp)	Het[4]
	1		c.1084G>C, p.(Glu362Gln)	Het[4]
	4	VHL	c.319C>G, p.(Arg107Gly)	Homo[6]
	3		c.239G>A, p.(Ser80Asn)	Homo[6]
	1		c.337C>G, p.(Arg113Gly)	Homo[6]
	1	EPAS1	c.1609G>A/c.1657dup, p.(Gly537Arg)/p.(Ala553Glyfs*58)	Het[4]
Remenyi et al. 2021 [27]	38	VHL		
	35	VHL	c.-195G>A	
Ediriwickrema et al. 2021 [28]	7	NGS of 45 genes		
	1	HFE	c.187C>G, p.(His63Asp) (HFE H63D)	Het[4]
	1	EGLN1	c.186G>C, p.(Glu62Asp)	Het[4]
		BMP6	c.427C>G/c.1098T>G, p.(Leu143Val)/p.(Ser366Arg)	Het[4]
		KDM6A	c.660A>T, p.(Lys220Asn)	Het[4]
	1	VHL	c.598C>T/c.376G>A, p.(Arg200Trp)/p.(Asp126Asn)	Het[4]
	1	SH2B3	c.1651C>T, p.(Arg551Trp)	Het[4]
	1	BPGM	c.273delC	
		EGLN1	whole gene duplication	
Mallik et al. 2022 [29]	1	NGS		
	1	HBB	c.92+5G>C/c.289C>G, p.(Leu97Val)	Het[4]
Lazana et al. 2021 [30]	1	Not specified		
	1	BPGM	c.260C>T, p.(Leu87Pro)	Homo[6]
